# Contrast-enhanced to non-contrast-enhanced image translation to exploit a clinical data warehouse of T1-weighted brain MRI

**DOI:** 10.1186/s12880-024-01242-3

**Published:** 2024-03-20

**Authors:** Simona Bottani, Elina Thibeau-Sutre, Aurélien Maire, Sebastian Ströer, Didier Dormont, Olivier Colliot, Ninon Burgos, APPRIMAGE Study Group

**Affiliations:** 1 Sorbonne Université, Institut du Cerveau - Paris Brain Institute - ICM, CNRS, Inria, Inserm, AP-HP, Hôpital de la Pitié-Salpêtrière, Paris, 75013 France; 2 Innovation & Données – Département des Services Numériques, AP-HP, Paris, 75013 France; 3 Hôpital Pitié Salpêtrière, Department of Neuroradiology, AP-HP, Paris, 75012 France; 4 Sorbonne Université, Institut du Cerveau - Paris Brain Institute - ICM, CNRS, Inria, Inserm, AP-HP, Hôpital de la Pitié-Salpêtrière, DMU DIAMENT, Paris, 75013 France

**Keywords:** Brain MRI, Clinical data warehouse, Image translation

## Abstract

**Background:**

Clinical data warehouses provide access to massive amounts of medical images, but these images are often heterogeneous. They can for instance include images acquired both with or without the injection of a gadolinium-based contrast agent. Harmonizing such data sets is thus fundamental to guarantee unbiased results, for example when performing differential diagnosis. Furthermore, classical neuroimaging software tools for feature extraction are typically applied only to images without gadolinium. The objective of this work is to evaluate how image translation can be useful to exploit a highly heterogeneous data set containing both contrast-enhanced and non-contrast-enhanced images from a clinical data warehouse.

**Methods:**

We propose and compare different 3D U-Net and conditional GAN models to convert contrast-enhanced T1-weighted (T1ce) into non-contrast-enhanced (T1nce) brain MRI. These models were trained using 230 image pairs and tested on 77 image pairs from the clinical data warehouse of the Greater Paris area.

**Results:**

Validation using standard image similarity measures demonstrated that the similarity between real and synthetic T1nce images was higher than between real T1nce and T1ce images for all the models compared. The best performing models were further validated on a segmentation task. We showed that tissue volumes extracted from synthetic T1nce images were closer to those of real T1nce images than volumes extracted from T1ce images.

**Conclusion:**

We showed that deep learning models initially developed with research quality data could synthesize T1nce from T1ce images of clinical quality and that reliable features could be extracted from the synthetic images, thus demonstrating the ability of such methods to help exploit a data set coming from a clinical data warehouse.

## Background

Clinical data warehouses, gathering hundreds of thousands of medical images from numerous hospitals, offer unprecedented opportunities for research. They can for example be used to develop and validate machine learning and deep learning algorithms for the computer-aided diagnosis of neurological diseases. However, they also pose important challenges, a major challenge being their heterogeneity. Neurological diseases can result in a variety of brain lesions that are each studied with specific magnetic resonance imaging (MRI) sequences. For example, T1-weighted (T1w) brain MR images enhanced with a gadolinium-based contrast agent are used to study lesions such as tumors, and T1w images without gadolinium are used to study neurodegenerative diseases.

Computer-aided diagnosis (CAD) systems for neurodegenerative diseases are more and more common in the clinic: they mainly include volumetric analysis, which can be used for the quantitative evaluation of brain atrophy [1–5]. Machine learning systems have been developed for research purposes, but their promising results for the differential diagnosis of neurodegenerative diseases indicate their potential application in the clinic [2, 6, 7]. These CAD systems, both based on volumetric analysis or on machine learning models, rely on features extracted from imaging data. Consequently, CAD systems are reliable only if input features are reliable. Additionally, features extracted from images must be homogeneous no matter the disease, otherwise a link could be established between MRI sequence and pathology, which would create bias. This is critical particularly in a clinical setting as differential diagnosis can be more challenging than in a research setting, as different diseases may co-exist.

Software tools such as SPM [8], ANTs [9] or FSL [10] have been widely used for feature extraction but they were largely validated using structural T1w MRI without gadolinium, to the best of our knowledge, and their good performance on images with gadolinium is thus not guaranteed. We are referring in particular to brain tissue segmentation algorithms: Unified Segmentation for SPM [11], FMRIB’s Automated Segmentation Tool (FAST) for FSL [12], and Atropos Multivar-EM Segmentation [13] and Multi-atlas methods [14] for ANTs. A solution could then be to convert contrast-enhanced T1w (T1ce) into non-contrast-enhanced T1w (T1nce) brain MRI before using such tools.

Deep learning has been widely used in the image translation domain. The goal of image translation is to learn a mapping between images of a source modality and images of a target modality, in order to convert an input image of the source modality into an image of the target modality. The U-Net and conditional generative adversarial networks (GANs) appear as the two most popular options. The U-Net was originally proposed for image segmentation [15, 16]: an encoder with convolutional and downsampling blocks is followed by a decoder with upsampling and convolutional layers. The skip connections linking the encoder and decoder blocks at the same level enable the reconstruction of fine-grained details, explaining the popularity of this architecture for image translation [17–24]. Conditional GANs consist of a generator, which may adopt the U-Net architecture, followed by a discriminator in charge of distinguishing synthetic from real images and challenging the generator so that it improves the quality of the generated images. The good results obtained with conditional GANs explain their wide use for image translation [25–34].

Both U-Net like models and conditional GANs have been proposed for diverse applications. Some aim to enhance the quality of the input images, for example by reducing noise in MRI [35–37] or positron emission tomography [38] images, or by performing super-resolution [25, 27, 39–41]. Other works aim to translate an image of a particular modality into another modality, such as an MRI into an X-ray computed tomography (CT) [19, 20, 24, 29, 17, 30] or a particular MRI sequence into another sequence [31–34]. The U-Net architecture has also been used for data harmonization, e.g. Deep-Harmony aims to homogenize the contrast between images coming from different sites [42]. 

Closer to our application, various deep learning models have been developed for the synthesis of images with gadolinium from images without gadolinium: they include reinforcement learning for liver MRI [43], or Gaussian mixture modeling for CT images [44]. As for the other image translation tasks, 3D U-Net like models have also been used to convert T1nce into T1ce images [45–47]. In two studies [45, 46], multimodal MRI sequences were used as input of the 3D U-Net that was trained and tested on patients with brain cancers. More specifically, the 3D U-Net proposed in [46] predicts patches of T1ce, while the one in [45] directly predicts the full 3D T1ce image. The residual attention U-Net described in [47] outputs synthetic T1nce that are used for the evaluation of cerebral blood volume in mice, instead of the real T1ce.

Our objective in this work was to evaluate how image translation models initially developed using research quality images could be used to exploit a highly heterogeneous data set from a clinical data warehouse by converting T1ce into T1nce images. We thus developed and compared different deep learning models that rely on typical architectures used in the medical image translation domain to convert T1ce into T1nce images. In particular, we implemented 3D U-Net like models with the addition of residual connections, attention modules or transformer layers. We also used these 3D U-Net like models in a conditional GAN setting. We trained and tested our models using 307 pairs of T1nce and T1ce images coming from a very large clinical data warehouse (39 different hospitals of the Greater Paris area). We first assessed synthesis accuracy by comparing real and synthetic T1nce images using standard metrics. We tested our models both on images of good or medium quality and on images of bad quality to ensure that deep learning models could generate accurate T1nce images no matter the quality of the input T1ce images. We then compared the volumes of gray matter, white matter and cerebrospinal fluid obtained by segmenting the real T1nce, real T1ce and synthetic T1nce images using SPM [11] in order to verify that features extracted from synthetic T1nce were reliable. Preliminary work was accepted for publication in the proceedings of the SPIE Medical Imaging 2022 conference [48]. Contributions specific to this paper include the development of additional models (a 3D U-Net like model with the addition of transformer layers, and three conditional GAN models using different 3D U-Net like models as generators and a patch-based discriminator) and an extended validation of the segmentation task with a deeper analysis of the tissue volume differences.

## Materials and methods

### Data set description

This work relies on a clinical data warehouse gathering all the T1w brain MR images of adult patients scanned in one of the 39 hospitals of the Greater Paris area (Assistance Publique-Hôpitaux de Paris [AP-HP]). The data were made available by the AP-HP data warehouse and the study was approved by the Ethical and Scientific Board of the AP-HP. According to French regulation, consent was waived as these images were acquired as part of the routine clinical care of the patients.

Among all the images of the clinical data warehouse, we selected only those referring to a 3D brain T1w MRI. This was done thanks to the manual selection by a neuro-radiologist of the DICOM header attributes (in particular the acquisition protocol, the series description and the body part) referring to a 3D brain T1w MRI [49].

In a previous work [49], we developed a quality control framework to identify images that are not proper T1w brain MRIs, to identify acquisitions for which gadolinium was injected, and to rate the overall image quality. The quality score assigned to each image is based on a three-level grade given to three different characteristics: contrast, motion and noise. A grade 0 corresponds to good contrast/no motion/no noise, a grade 1 to medium contrast/some motion/some noise and a grade 2 to bad contrast/severe motion/severe noise. If at least one of the characteristics has a grade of 2, the image is labeled with a low quality score. If at least one of the characteristics has a grade of 1 and no characteristic has a grade of 2, the image is labeled with a medium quality score. If all characteristics have a grade of level 0, the image is labeled as good quality. We manually annotated 5500 images (out of a batch of 9941 images that were available, excluding images with less than 40 DICOM slices) to train and test convolutional neural network (CNN) classifiers. The graphical interface used to manually annotate the images is publicly available (https://github.com/SimonaBottani/Quality_Control_Interface).

**Fig. 1:**
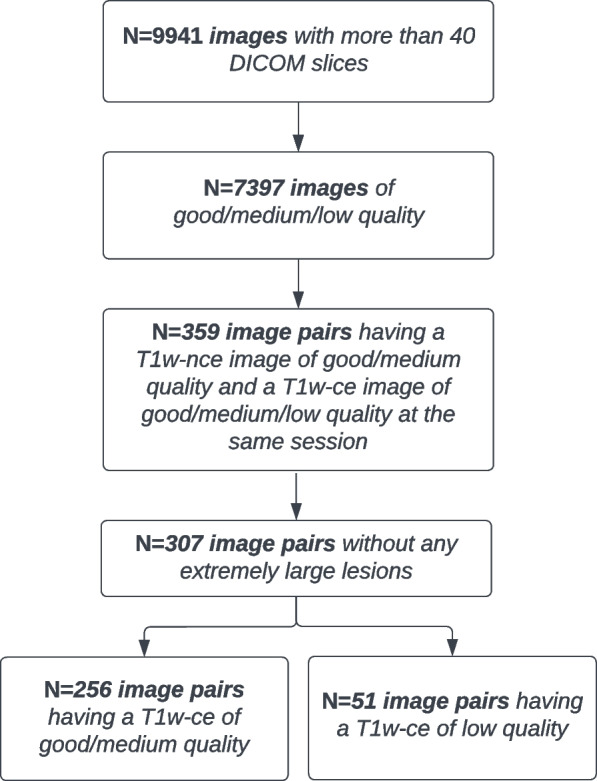
Description of the different steps for the selection of the data set

The data set used in this work is composed of 307 pairs of 3D T1ce and T1nce images that were extracted from the batch of 9941 images made available by the AP-HP data warehouse. Their resolution ranges from 0.9 to 1.2 mm. We first selected all the images of low, medium and good quality, excluding images that were not proper T1w brain MRI [49], resulting in 7397 images. This selection was based on manual quality control for 5500 images and on automatic quality control for the remaining 4441 images [49]. In the same way, the presence or absence of gadolinium-based contrast agent was manually noted for 5500 images, while it was obtained through the application of a CNN classifier for the remaining 4441 images. We then considered only patients having both a T1ce and a T1nce image at the same session, with a T1nce image of medium or good quality. Finally, to limit heterogeneity in the training data set, we visually checked all the images and excluded 52 image pairs that were potential outliers because of extremely large lesions (i.e., lesions that substantially altered surrounding brain tissues). Among the selected images, 256 image pairs were of medium and good quality, and 51 image pairs had a T1ce of low quality and a T1nce of good or medium quality. In total the data set comprises 614 images: 534 images were acquired at 3 T and 80 at 1.5 T, 556 images were acquired with a Siemens machine (with seven different models) and 58 with a GE Healthcare machine (with five different models). The workflow in Fig. 1 describes the selection of the data set for our work.

### Image preprocessing

All the images were organized following the Brain Imaging Data Structure (BIDS) specification [50]. We applied the following pre-processing using the t1-linear pipeline of the open-source software platform Clinica [51], which is a wrapper of the ANTs software [9]. Bias field correction was applied using the N4ITK method [52]. An affine registration to MNI space was performed using ANTs [53]. The registered images were further rescaled based on the min and max intensity values, and cropped to remove background resulting in images of size 169$\times$208$\times$179, with 1 mm isotropic voxels [54]. Finally all the images were resampled to have a size of 128$\times$128$\times$128 using trilinear interpolation in Pytorch.

### Network architecture

To generate T1nce from T1ce images, both 3D U-Net like models and conditional GANs were developed and compared. The code used to implement all the architectures and perform the experiments is openly available (https://github.com/SimonaBottani/image_synthesis).

#### 3D U-Net like structures

**Fig. 2:**
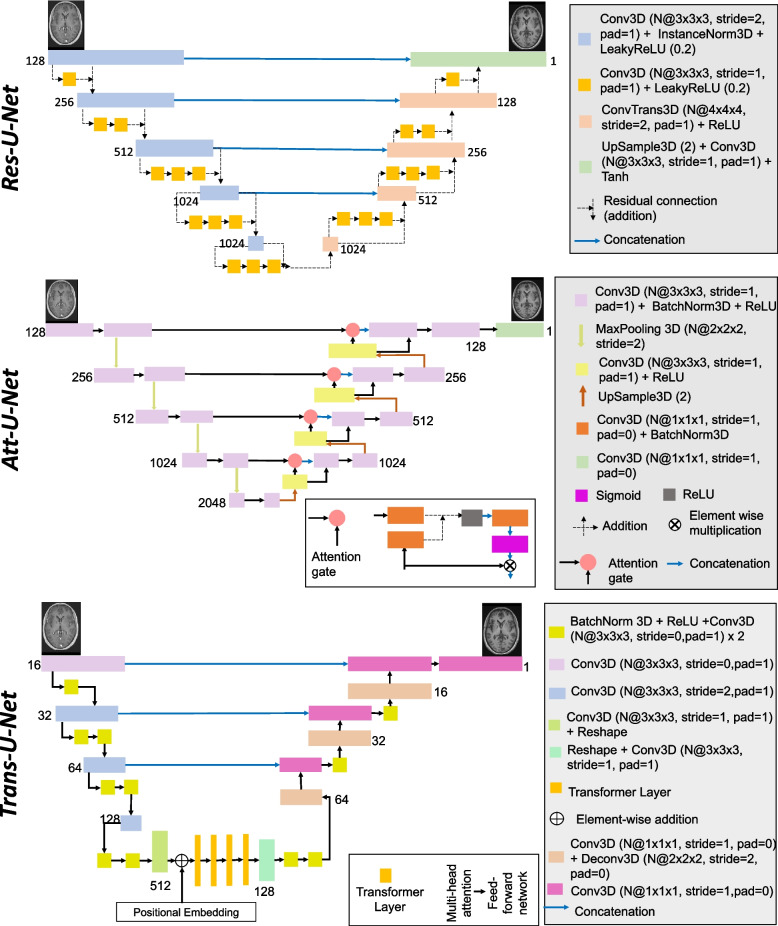
Architectures of the proposed 3D U-Net like models. The models take as input a real T1nce image of size 128$\times$128$\times$128 and generate a synthetic T1nce of size 128$\times$128$\times$128. *Res-U-Net*: images pass through five descending blocks, each one followed by a residual module, and then through four ascending blocks and one final layer. *Att-U-Net*: images pass through five descending blocks and then through four ascending blocks and one final layer. One of the inputs of each ascending block is the result of the attention gate. *Trans-U-Net*: images pass through four descending blocks, four transformer layers and four ascending layers. All the parameters such as kernel size, stride, padding, size of each feature map (N) are reported

We implemented three models derived from the 3D U-Net [15, 16]: a 3D U-Net with the addition of residual connections (called *Res-U-Net*) [45, 55], a 3D U-Net with the addition of attention mechanisms (called *Att-U-Net*) [56], and a 3D U-Net with both transformer and convolutional layers (called *Trans-U-Net*) [57] to study how already developed architectures could be adapted to our context, i.e. synthesis from highly heterogeneous images of clinical quality. The U-Net structure allows preserving the details present in the original images thanks to the skip connections [15] and has shown good performance for image-to-image translation [17–24]. Here we detail the three architectures, which are also shown in Fig. 2.

##### Res-U-Net

The *Res-U-Net* we implemented is based on the architecture first proposed by [55] and later used by [45]. The five descending blocks are composed of 3D convolutional layers followed by an instance normalization block and a LeakyReLU (negative slope coefficient $\alpha =0.2$). The four ascending blocks are composed of transposed convolutional layers followed by a ReLU. The final layer is composed of an upsample module (factor of 2), a 3D convolutional block and a hyperbolic tangent module. Each descending or ascending block is followed by a residual module, which can vary from one to three blocks composed of a 3D convolutional layer and a LeakyReLU ($\alpha =0.2$). Residual blocks were introduced to avoid the problem of the vanishing gradients in the training of deep neural networks [58]: they ease the training since they improve the flow of the information within the network.

##### Att-U-Net

We implemented the *Att-U-Net* relying on the work of [56]. In this architecture, the five descending blocks are composed of two blocks with a 3D convolutional layer followed by a batch normalization layer and a ReLU. They are followed by four ascending blocks. Each ascending block is composed of an upsample module (factor of 2), a 3D convolutional layer followed by a ReLU, an attention gate and two 3D convolutional layers followed by a ReLU. The attention gate is composed of two 3D convolutional layers, a ReLU, a convolutional layer and a sigmoid layer. Its objective is to identify only salient image regions: the input of the attention gate is multiplied (element-wise multiplication) by a factor (in the range 0–1) resulting from the training of all the blocks of the networks. In this way it discards parts of the images that are not relevant to the task at hand.

##### Trans-U-Net

The *Trans-U-Net* was implemented by [57] (who called the model *TransBTS*). They proposed a 3D U-Net like structure composed of both a CNN and a transformer. The CNN is used to produce an embedding of the input images in order not to loose local information across depth and space. The features extracted by the CNN are the input of the transformer whose aim is to model the global features. The descending blocks are composed of four different blocks, each being composed of a 3D convolutional layer and one, two or three blocks composed of a batch normalization layer, a ReLU and another 3D convolutional layer. The model is then composed of four transformer layers, after a linear projection of the features. Each transformer layer is itself composed of a multi-head attention block and a feed forward network. The four ascending blocks are composed of a 3D convolutional layer and one or two blocks with a batch normalization layer, a ReLU, a 3D convolutional layer followed by a 3D deconvolutional layer. The final layer is composed of a 3D convolutional layer and a soft-max layer.

For the three 3D U-Net like models we used the same training parameters. We used the Adam optimizer, the $L_1$ loss, a batch size of 2 and trained during 300 epochs. The model with the best loss, determined using the training set, was saved as final model. We relied on Pytorch for the implementation.

#### Conditional GANs

Generative adversarial networks (GANs) were first introduced by [59]. They are generative deep learning models composed of two elements: a generator for synthesizing new examples and a discriminator for classifying whether examples are real, i.e. the original ones, or fake, i.e. synthesized by the generator. Conditional GANs (cGANs) [60] are a variant of GANs where the generator and the discriminator are conditioned by the true samples. They can only be used with paired data sets.

We propose three different cGAN models that differ in the architecture of the generators, which correspond to the three architectures presented above. The discriminator is the same for all the cGANs: it is a 3D patch CNN, first proposed by [61] and used in the medical image translation domain [62, 63]. Its aim is to classify if each pair of patches contains two real images, or a real and a fake image. The advantages of working with patches is that the discriminator focuses on the details of the images and the generator must improve them to fool the discriminator.

Our discriminator is made of four blocks: the first three blocks are composed of a 3D convolutional layer followed by a LeakyReLU (negative slope coefficient $\alpha =0.2$), and the last block is composed of a 3D convolutional layer and a 3D average pooling layer. From images of size 128$\times$128$\times$128, we created eight patches of size 64$\times$64$\times$64 with a stride of 50.

For the training of the discriminator we used the least square loss as proposed in [64] in order to increase the stability, thus avoiding the problem of vanishing gradients that occurs with the usual cross-entropy loss. Stability of the training was also improved using soft labels: random numbers between 0 and 0.3 represented real images and random numbers between 0.7 and 1 represented fake images.

The total loss of the cGANs combines

- the loss of the generator composed of the sum of the $L_1$ loss (i.e. pixel-wise absolute error) computed between the generated and true images, and the least square loss computed between the predicted probabilities of the generated images and positive labels
  1 $$\begin{aligned} L_{G} = -\log \left[ p(\text {synthetic T1nce}) \right] + L_1(\text {T1nce}, \text {synthetic T1nce}) \end{aligned}$$
  with *p*(*X*) the probability returned by the discriminator that the image *X* is real.
- the loss of the discriminator composed of the mean of the least square loss computed between the predicted probabilities of the true images and positive labels, and the least square loss computed between the predicted probabilities of the generated images and negative labels
  2 $$\begin{aligned} L_{D} = -0.5\log \left[ p(\text {T1nce}) \right] -0.5\log \left[ 1- p(\text {synthetic T1nce}) \right].\end{aligned}$$

At first, both the generators and discriminators were pretrained separately. The adversarial nature of GANs makes their training time consuming. In our experimental setting, constrained by the computational resources available within the clinical data warehouse, we have found out that using a pretrained generator and discriminator, each with an already established good performance, can stabilize the training of the cGAN. In particular, we have seen that it can prevent the vanishing gradient effect in the discriminator. This was observed experimentally, but other works have described the advantages of pretrained models [65–67]. Regarding each generator, we reused the best model obtained previously. The discriminators were pretrained for the recognition of real and fake patches (fake images were obtained from each pretrained generator). The generators and discriminators were then trained together. The generator models with the best loss, determined using the training set, were saved as final models. Note that the batch size was set to 1 due to limited computing resources.

### Experiments and validation measures

The experiments relied on 307 pairs of T1ce and T1nce images. We randomly selected 10% of the 256 image pairs of medium and good quality for testing (data set called Test_good_), the other 230 image pairs being used for training. Only images of good and medium quality were used for training to ensure that the model focuses on the differences related to the presence or absence of gadolinium, and not to other factors. The remaining 51 image pairs with a T1ce of low quality and a T1nce of good or medium quality were used only for testing (data set called Test_low_).

#### Synthesis accuracy

Image similarity was evaluated using the mean absolute error (MAE), peak signal-to-noise ratio (PNSR) and structural similarity (SSIM) [68]. The MAE is the mean of each absolute value of the difference between the true pixel and the generated pixel and PSNR is a function of the mean squared error: these two metrics allow a direct comparison between the synthetic image and the real one. The SSIM aims to measure quality by capturing the similarity of images, it is a weighted combination of the luminance, contrast and structure. For the MAE, the minimum value is 0 (the lower, the better), for PSNR the maximum value is infinite (the higher, the better) and for SSIM the maximum value is 1 (the higher, the better). We calculated these metrics both between the real and synthetic T1nce images and between the real T1nce and T1ce images (as reference). These metrics were calculated within the brain as this region is the main focus of our evaluation. A brain mask was obtained for each subject by skull-stripping the T1nce and T1ce images using HD-BET [69] and computing the union of the two resulting brain masks.

#### Segmentation fidelity

Our goal is to obtain gray matter (GM), white matter (WM) and cerebrospinal fluid (CSF) segmentations from T1ce images using widely-used software tools that are consistent with segmentations obtained from T1nce images. We thus assessed segmentation consistency by analyzing the tissue volumes resulting from the segmentations, which are important features when studying atrophy in the context of neurodegenerative diseases. We used the algorithm proposed in SPM [11] but these features can be obtained with commercial tools, such as NeuroreaderTM, volBrain, NeuroQuant or Inbrain, and used in a clinical setting [1–6].

The volumes of the different tissues were obtained as follows. At first, synthetic T1nce images were resampled back to a size of 169$\times$208$\times$179 using trilinear interpolation in Pytorch so that real and synthetic images have the same grid size. We processed the images using the t1-volume-tissue-segmentation pipeline of Clinica [51, 70]. This wrapper of the Unified Segmentation procedure implemented in SPM [11] simultaneously performs tissue segmentation, bias correction and spatial normalization. Once the probability maps were obtained for each tissue, we computed the maximum probability to generate binary masks and we multiplied the number of voxels by the voxel dimension to obtain the volume of each tissue. We calculated both the absolute volume difference (AVD) and the volume difference (VD) for each tissue between the real T1ce or synthetic T1nce and the real T1nce as follows:

3a $$\begin{aligned} \text {AVD}&= \frac{|V^I_t - V^J_t |}{TIV^I} \times TIV,\quad\end{aligned}$$

3b $$\begin{aligned} \text {VD}&= \frac{ V^I_t - V^J_t}{TIV^I} \times TIV,\quad\end{aligned}$$

where $V^I_t$ is the volume of tissue *t* extracted from the real T1nce image *I*, $V^J_t$ is the volume of tissue *t* extracted from image *J*, *J* being the synthetic T1nce or real T1ce image. $TIV^I$ corresponds to the total intracranial volume (sum of the gray matter, white matter and cerebrospinal fluid volumes) obtained from the real T1nce image *I* and *TIV* corresponds to the average total intracranial volume computed across the two test sets. The multiplication by the average total intracranial volume (TIV) aims at obtaining volumes (in cm^3^) rather than fractions of the TIV of each subject, which is easier to interpret. Since this is a multiplication by a constant, it has no impact on the results. To assess whether the tissue volumes presented a statistically significant difference in terms of AVD depending on the images they were obtained from, we performed paired t-tests using Bonferroni correction for multiple comparisons.

In addition, we compared the binary tissue maps extracted from the real T1ce or synthetic T1nce image to those extracted from the real T1nce using the Dice score.

## Results

We report results for the proposed generator-only 3D U-Net like models and cGANs trained on 230 image pairs of good and medium quality, and tested on Test_good_ and Test_low_ obtained from a clinical data set.

**Fig. 3:**
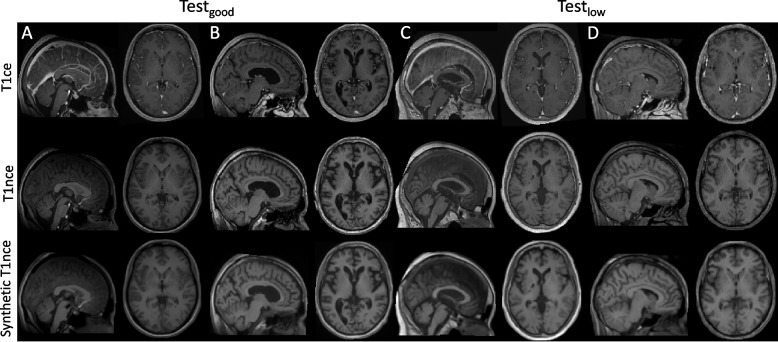
Examples of real T1ce (top), real T1nce (middle) and synthetic T1nce obtained with the *cGAN Att-U-Net* model (bottom) images in the sagittal and axial planes. Images of patients A and B belong to Test_good_ (left) while images of patients C and D belong to Test_low_ (right)

Examples of synthetic T1nce images obtained with the *cGAN Att-U-Net* model together with the real T1ce and T1nce images are displayed in Fig. 3. Images of patients A and B belong to Test_good_ while images of patients C and D belong to Test_low_. We note the absence of contrast agent in the synthetic T1nce, while it is clearly visible in the sagittal slice of the T1ce (particularly visible for patients A and C) and that the anatomical structures are preserved between the synthetic and real T1nce, even in the case of a disease (as for patient B). We also note that contrast between gray and white matter is preserved in the synthetic T1nce (particularly visible for patients B and D). For Test_low_, the contrast seems improved in the synthetic compared with the real T1ce image (especially for patient D). This results is not surprising as the networks were trained with images of medium or good quality, which will have on average a better contrast than images of low quality.

### Synthesis accuracy

Table 1 reports the image similarity metrics obtained for the two test sets within the brain region. We computed these metrics to assess the similarity between real and synthetic T1nce images, but also between T1nce and T1ce images to set a baseline. We observe that, for all models, the similarity is higher between real and synthetic T1nce images than between T1nce and T1ce images according to all three metrics on both test sets. The differences observed in terms of MAE, PSNR and SSIM between the baseline and each image translation approach are statistically significant (corrected *p*-value < 0.05 according to a paired t-test corrected for multiple comparisons using the Bonferroni correction).

**Table 1:** MAE, PSNR and SSIM obtained on the two independent test sets with various image quality. For each metric, we report the average and standard deviation across the corresponding test set. We compute the metrics for both real T1ce and synthetic T1nce in relation to the real T1nce, and so within the brain region

Test set	Compared images	Model	MAE (%)	PSNR (dB)	SSIM
Test_good_	T1nce / T1ce	-	4.14 ± 1.59	23.03 ± 2.83	0.90 ± 0.05
	T1nce / Synthetic T1nce	*Res-U-Net*	3.06 ± 1.50	26.89 ± 4.30	0.95 ± 0.04
		*Att-U-Net*	2.73 ± 1.69	29.07 ± 4.53	0.96 ± 0.05
		*Trans-U-Net*	2.80 ± 1.42	28.00 ± 4.13	0.96 ±0.04
		*cGAN Res-U-Net*	3.47 ± 1.59	23.89 ± 4.30	0.95 ± 0.04
		*cGAN Att-U-Net*	2.69 ± 1.68	28.89 ± 4.44	0.97 ± 0.05
		*cGAN Trans-U-Net*	2.86±1.59	28.00 ±4.32	0.96 ± 0.04
Test_low_	T1nce / T1ce	-	3.71 ± 1.99	24.20 ± 3.85	0.91 ± 0.06
	T1nce / Synthetic T1nce	*Res-U-Net*	2.93 ± 1.77	26.71 ± 4.32	0.95 ± 0.05
		*Att-U-Net*	2.89 ± 1.85	27.15 ± 4.57	0.95 ± 0.05
		*Trans-U-Net*	2.98 ± 1.89	26.71 ± 4.38	0.94 ± 0.05
		*cGAN Res-U-Net*	3.20 ± 1.96	26.20 ± 4.42	0.93 ± 0.05
		*cGAN Att-U-Net*	2.86 ± 1.83	27.12 ± 4.50	0.95 ± 0.05
		*cGAN Trans-U-Net*	2.97 ± 1.83	26.68 ± 4.40	0.94 ± 0.05

Among the generator-only 3D U-Net like models, the *Att-U-Net* performed slightly better than the others, both for Test_good_ (mean MAE: 2.73%, PSNR: 29.07 dB, SSIM: 0.96) and Test_low_ (mean MAE: 2.89%, PSNR: 27.18 dB, SSIM: 0.95). The performance of the cGANs were comparable to their counterparts composed only of the generator. *cGAN Att-U-Net* had a lower MAE for both test sets (mean MAE: 2.69% for Test_good_ and mean MAE: 2.86% for Test_low_). There was no statistically significant difference observed, no matter the synthesis accuracy measure, between *cGAN Att-U-Net*, the best performing model according to the MAE, and the other approaches for both test sets (corrected *p*-value > 0.05). For further validation we kept only the generator-only *Att-U-Net* and *cGAN Att-U-Net*.

### Segmentation fidelity

**Fig. 4:**
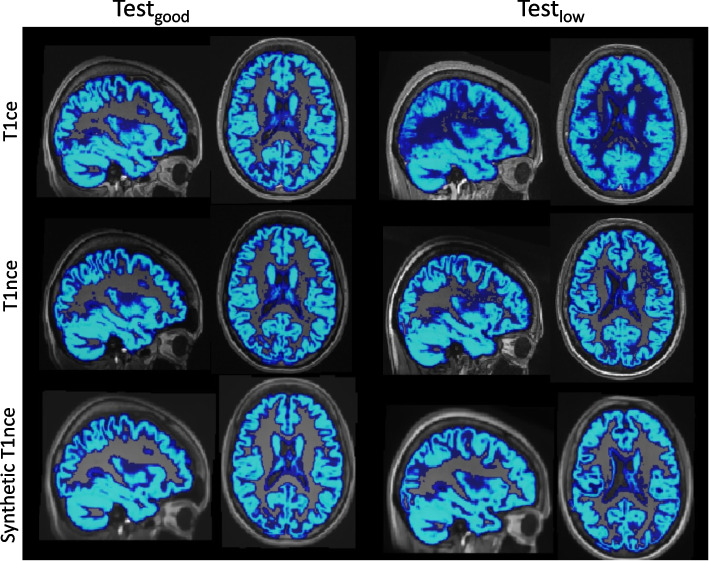
Example of the probability gray matter maps obtained from T1ce (top), T1nce (middle) and synthetic T1nce (*cGAN Att-U-Net* model, bottom) images from Test_good_ (left) and Test_low_ (right)

Examples of probability gray matter maps obtained from T1ce, T1nce and synthetic T1nce images are displayed in Fig. 4. Compared with the T1ce images, the gray matter maps obtained from the synthetic T1nce better resembles that extracted from the T1nce, especially for Test_low_.

Absolute volume differences (AVD) obtained between T1nce and T1ce images and between T1nce and synthetic T1nce images (obtained with the generator-only *Att-U-Net* model and the *cGAN Att-U-Net*) for GM, WM and CSF are reported in Table 2. For both test sets and all tissues, the absolute volume differences are smaller between T1nce and synthetic T1nce images than between T1nce and T1ce images for the two models. Using the generator-only *Att-U-Net* on Test_good_, absolute volume differences of GM and CSF between T1nce/T1ce and T1nce/Synthetic T1nce are statistically significantly different (corrected *p*-value < 0.01 according to a paired t-test corrected for multiple comparisons using the Bonferroni correction), while on Test_low_ absolute volume differences of all the tissues are statistically significantly different (corrected *p*-value < 0.01). Using the *cGAN Att-U-Net* model, absolute volume differences of all the tissues are statistically significantly different (corrected *p*-value < 0.01) for both test sets. This means that there is an advantage in using synthetic T1nce images rather than T1ce images, no matter the model used for the synthesis: segmentation of GM, CSF and WM is more reliable since closer to the segmentation of the tissues in the real T1nce.

**Table 2:** Absolute volume difference (mean ± standard deviation in cm^3^) between T1nce and T1ce images and between T1nce and synthetic T1nce images (obtained with the generator-only *Att-U-Net* and *cGAN Att-U-Net* models) for gray matter, white matter and cerebrospinal fluid (CSF). * indicates that the absolute volume difference between T1nce and synthetic T1nce images is statistically significantly different from that of the baseline (corrected *p*-value <0.01) according to a paired t-test corrected for multiple comparisons using the Bonferroni correction

	Compared images	Model	Test_good_ [cm^3^]	Test_low_ [cm^3^]
Gray matter	T1nce / T1ce	-	26.68 ± 15.92	49.63 ± 49.38
	T1nce / Synthetic T1nce	*Att-U-Net*	10.36 ± 6.98 *	19.61 ± 29.54 *
		*cGAN Att-U-Net*	9.24 ± 6.10 *	19.67 ± 28.32 *
White matter	T1nce / T1ce	-	10.81 ± 3.71	25.36 ± 27.73
	T1nce / Synthetic T1nce	*Att-U-Net*	7.79 ± 5.87	13.95 ± 24.74 *
		*cGAN Att-U-Net*	6.40 ± 4.43 *	14.49 ± 21.06 *
CSF	T1nce / T1ce	-	61.62 ± 34.61	69.55 ± 37.77
	T1nce / Synthetic T1nce	*Att-U-Net*	13.37 ± 10.18 *	12.25 ± 7.72 *
		*cGAN Att-U-Net*	18.27 ± 17.20 *	17.10 ± 18.45 *

**Fig. 5:**
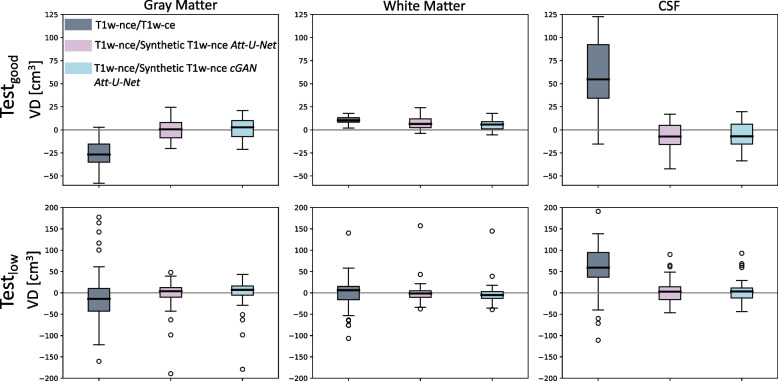
Volume differences (VD) in cm^3^ between T1nce and T1ce images and between T1nce and synthetic T1nce images (obtained with the generator-only *Att-U-Net* and the *cGAN Att-U-Net* models) for gray matter (left), white matter (middle) and cerebrospinal fluid (CSF, right) for both Test_good_ (top) and Test_low_ (bottom)

Volume differences (VD) computed between T1nce and T1ce images and between T1nce and synthetic T1nce images (obtained with the generator-only *Att-U-Net* and *cGAN Att-U-Net*) for GM, WM and CSF are reported in Fig. 5. We observe that volumes extracted from T1ce images tend to be over-estimated (GM) or under-estimated (CSF) and that most of these biases disappear when tissues are extracted from synthetic T1nce images (mean VD closer to 0).

The Dice scores obtained when comparing the GM, WM and CSF segmentations between T1nce and T1ce images and between T1nce and synthetic T1nce images (obtained with the generator-only *Att-U-Net* and the *cGAN Att-U-Net*) are displayed in Table 3. We observe that for both gray and white matter, the Dice scores are similar between T1nce and T1ce or synthetic T1nce images, while for CSF higher Dice scores are obtained using synthetic T1nce images.

**Table 3:** Dice scores obtained when comparing the gray matter, white matter and cerebrospinal fluid (CSF) segmentations between T1nce and T1ce images and between T1nce and synthetic T1nce images (obtained with the generator-only *Att-U-Net* and the *cGAN Att-U-Net*)

	Compared images	Model	Test_good_	Test_low_
Gray matter	T1nce / T1ce	-	0.88 ± 0.02	0.77 ± 0.12
	T1nce / Synthetic T1nce	*Att-U-Net*	0.87 ± 0.02	0.81 ± 0.07
		*cGAN Att-U-Net*	0.87 ± 0.02	0.81 ± 0.07
White matter	T1nce / T1ce	-	0.93 ± 0.01	0.85 ± 0.10
	T1nce / Synthetic T1nce	*Att-U-Net*	0.90 ± 0.02	0.86 ± 0.04
		*cGAN Att-U-Net*	0.91 ± 0.02	0.86 ± 0.03
CSF	T1nce / T1ce	-	0.63 ± 0.10	0.62 ± 0.10
	T1nce / Synthetic T1nce	*Att-U-Net*	0.80 ± 0.05	0.78 ± 0.07
		*cGAN Att-U-Net*	0.80 ± 0.05	0.78 ± 0.07

## Discussion

The use of clinical images for the validation of computer-aided diagnosis systems is still largely unexplored. One of the obstacles lies in the heterogeneity of the data acquired in the context of routine clinical practice. Post-acquisition homogenization is crucial because, contrary to research data, no strict acquisition protocols, that would ensure a certain homogeneity among the images, exist for clinical data. Heterogeneity originates from the fact that images are acquired with different scanners at different field strengths during a large period of time and because patients may suffer from a large variety of diseases. Homogenization of clinical data sets of 3D T1w brain MRI, and consequently of the features extracted from them, is an important step for the development of reliable CAD systems. Indeed, when training a CAD system, the algorithms must not be affected by the data set variations even though clinical images may greatly vary.

A source of heterogeneity among clinical data sets is the fact that they contain a mix of images acquired with and without gadolinium-based contrast agent. In our case, among the 7397 proper T1w brain images made available by the AP-HP data warehouse out of a batch of 9941 images, 59% of the images were contrast-enhanced [49]. As a first step towards the homogenization of this data set, we thus proposed a framework to convert T1ce images into T1nce images using deep learning models. The choice to synthesize T1nce images from T1ce images was constrained by the fact that software tools for feature extraction in the neuroimaging community were developed for T1nce MRI. To the best of our knowledge, none of these tools has largely been applied to the extraction of features from T1ce MRI data and their performance in this scenario is thus mostly unknown.

The contribution of our work consists in the development and validation of deep learning models (generator-only U-Net models and conditional GANs) for the translation of T1ce to T1nce images coming from a clinical data warehouse. We compared three 3D U-net models differentiated by the addition of residual modules, of attention modules or of transformer layers, used as simple generators and also within a conditional GAN setting with the addition of a patch-based discriminator. These models have widely been used for the image translation of medical images [71, 72], but to the best of our knowledge, their application to clinical data has not been proven yet. The proposed models were trained using 230 image pairs and tested on two different test sets: 26 image pairs had both a T1nce and T1ce of good or medium quality and 51 image pairs had a T1nce of good or medium quality and a T1ce of bad quality. Having two test sets of different qualities is a key point since we are dealing with a real clinical heterogeneous data set (e.g., acquisitions from 12 scanner models), where images of low quality, corresponding in majority to T1ce images with a low contrast, may represent 30% of the data [49].

We first assessed the similarity between real and synthetic T1nce images and between real T1nce and T1ce images using three similarity metrics, MAE, PSNR and SSIM. We showed that the similarity between real and synthetic T1nce images was higher than the similarity between real T1nce and T1ce images according to all the metrics, no matter the models used nor the quality of the input image. The synthesis accuracy obtained with the models evaluated was of the same order as the one reached in recent works on non-contrast-enhanced to contrast-enhanced image translation [45, 46]. The performance of all the models was equivalent (no statistically significant difference observed), meaning that all were able to synthesize T1nce images. Slightly better performance was reached with the addition of attention modules (generator-only *Att-U-Net* and *cGAN Att-U-Net* models), and these models were thus further evaluated. Note that the image similarity metrics were computed within the brain region, as this was the main focus of our work, and that another conclusion could have been reached when computing these metrics for the whole head.

In the second step of the validation, we assessed the similarity of features extracted from the different images available using a widely adopted segmentation framework known for its robustness, SPM [8, 11]. For the evaluation of the segmentation, we reported the absolute volume difference, the volume difference and the Dice scores. We showed that the absolute volume differences of GM, WM and CSF were larger between real T1nce and T1ce images than between real and synthetic T1nce images (statistically significant difference most of the times, systematically for GM which is the main feature when studying atrophy in neurodegenerative diseases). This confirms the hypothesis that gadolinium-based contrast agent may alter the contrast between the different brain tissues, making features extracted from such images with standard segmentation tools, here SPM [8, 11], unreliable. At the same time, we validated the suitability of the synthetic images since their segmentation was consistent with those obtained from real T1nce images as the absolute volume differences were small. The fact that the differences between the volumes extracted from the real and synthetic T1nce images are relatively close to zero show that the tissue volumes are not systematically under- or over-estimated when extracted from the synthetic images. When analyzing the Dice scores in the gray matter and white matter, we observed that they are mostly equivalent when computed between real T1nce and T1ce or between real and synthetic T1nce. The improvement brought by the synthetic T1nce is only observed in the CSF. This is slightly different from what was observed when analyzing the absolute volume differences. This is due to the fact that the Dice score is normalized and that we report the volume difference in cm^3^. Nevertheless, we mainly focused on the analysis of the volume differences because the goal of our work is to use volumetric features as input for machine learning or deep learning models for computer-aided diagnosis. Future work could consist in extending the volumetric analysis to subcortical regions. It could also consist in further evaluating our approach on surface-based features such as cortical thickness.

Even though the synthetic T1nce images enable the extraction of reliable features, their quality could still be improved. Many constraints exist when working with data from a clinical data warehouse. One is the fact that these data are accessible only through a closed environment provided by the IT department of the AP-HP as described in [73]. Limitations in computational resources and storage space make training deep learning models difficult, which limits the experiments that can be performed to find the optimal model. In particular, in order to have as much data as possible for training, we decided to split our data set in just a training and two test sets for this work. With more data and more computational resources, a proper split into training, validation and test sets would have been more suitable. The proposed models could be improved by better optimizing the hyperparameters (such as the learning rate or the size of the kernels), adding a perceptual loss when training the conditional GANs [74] or adding more layers in the patch-based discriminator. Other architectures could also be explored. We have restricted our work to conditional GANs, which need paired data to be trained, but we could exploit more data working with cycle GANs [75] as they can deal with unpaired data.

In any case, several steps remain to be performed before using synthetic T1nce images for the differential diagnosis of neurological diseases in a clinical setting. First, the preprocessing steps should be minimized. This would for example imply using images in their native space instead of images spatially normalized to the MNI space as we did in this work to ease the evaluation of the approach. In addition, the performance of CAD systems trained with a mix of real T1nce and T1ce images should be compared with the performance of CAD systems trained with a mix of real and synthetic T1nce images. To prevent introducing a correlation between image properties (e.g. smoothness) and pathology, which would bias the classification performance, it may be necessary to also feed the real T1nce images to the neural network and use the resulting images as inputs of the CAD system, as suggested in [42]. Furthermore, heterogeneity within a clinical data set can arise from other sources, such as the use of different MRI scanner machines or different acquisition parameters. Future works should study their influence and propose models to achieve a more general homogenization, as proposed in [76]. Thanks to these improvements, the application of the proposed homogenization framework would not be limited to differential diagnosis but could be extended to the study of disease progression, which requires capturing more subtle volume differences.

## Conclusions

Clinical data warehouses offer fantastic opportunities for computer-aided diagnosis of neurological diseases but their heterogeneity must be reduced to avoid biases. As a first step to homogenize such a large clinical data set, this work proposed to convert images acquired after the injection of gadolinium into non-contrast-enhanced images using 3D U-Net models and conditional GANs. Validation using standard image similarity measures demonstrated that the similarity between real and synthetic T1nce images was higher than between real T1nce and T1ce images for all the models compared. We also showed that features extracted from the synthetic images (GM, WM and CSF volumes) were closer to those obtained from the T1nce brain MR images (considered as reference) than the original T1ce images. These results demonstrate the ability of deep learning methods to help exploit a data set coming from a clinical data warehouse.

## Data Availability

Accessing the data is possible with the following procedure. A detailed project must be submitted to the Scientific and Ethics Board of the AP-HP. If the project participants are external to AP-HP, they have to sign a contract with the Clinical Research and Innovation Board (Direction de la Recherche Clinique et de l’Innovation). The project must include the goals of the research, the different steps that will be pursued, a detailed description of the data needed, of the software tools necessary for the processing, and a clear statement of the public health benefits. Once the project is approved, the research team is granted access to the Big Data Platform (BDP), which was created by a sub-department of the IT of the AP-HP. The BDP is a platform internal to the AP-HP where data are collected and that external users can access to perform all their analyses, in accordance with the CNIL regulation. It is strictly forbidden to export any kind of data and each user can access only a workspace that is specific to their project. Each person of the research team can access the BDP with an AP-HP account after two-factor authentication. If the research team includes people that are not employed by the AP-HP, a temporary account associated to the project is activated.
